# Extra Supplement—The Nursing Mirror

**Published:** 1889-08-24

**Authors:** 


*The Hospital,*
AUGUST 24, 1889.

### The Nursing Mirror.

*Extra Supplement*

All Contributions for this Supplement should be addressed to the Editor, THE HOSPITAL, 139-140, Salisbury Court, Fleet Street, London, E.C., and should have the word "Nursing" plainly written in left-hand top corner of the envelope.

### En Passant.

THE DOLL COMPETITION.—In reply to different correspondents, we beg to state that a set of dolls to represent any hospital staff need not consist of any definite number. Where there are only two uniforms worn, as is the case, we believe, at Leamington and elsewhere, two dolls will form the set; where there are four uniforms, as at Guy's, four dolls will form the set. The desire is to have every hospital and institution represented at the show, even those district nursing institutions which only boast one uniform.

LADY DENTISTS.—We heard once of a district nurse in a country parish who included tooth-extracting amongst her many duties, but we little thought dentists of the gentler sex would rise so high in the profession as is indicated by the standing of the young woman who has just been graduated from the Boston Dental College. According to the announcement of the dean, she stood No. 1 in a class of between thirty and forty, and in the race she has run she was so far ahead of her classmates that she could hardly hear the tread of the fellow next behind her. The dentist's chair is not exactly a synonym for everything that is comfortable and inviting, but the presence of a gentle woman operator promises to detract from its terrors.

THE MIDWIVE'S INSTITUTE.—In last week's *Lancet* appeared a letter from the Secretary of the Midwive's Institute and Trained Nurses' Club asking for medical books. This institution endeavours to give all possible help to those midwives who are preparing themselves for the Obstetrical Society's examination, and all works on midwifery are invaluable to these women, many of whom have very small means, and consequently their education is a very severe strain upon their incomes. Frequent requests come from country members to the librarian to send them the different standard works for their studies, and the institute cannot meet the demand, as they have a very limited supply of these books, although some medical men have generously presented their works. Though most large hospitals have medical libraries for the nurses, there are a vast number of infirmaries and institutions where neither books of instruction nor amusement are provided for nurses, and to these last the library at 15, Buckingham-street must be a tremendous advantage.

REMINISCENCES OF DR. ROGERS.—The late Dr. Rogers left behind him a book describing the state of workhouses when he first became medical officer. Dr. Rogers had not been many months medical officer to the Strand when he found that his pauper nurses were frequently under the influence of drink, and that, too, in the forenoon, the explanation of this being that the master of the workhouse, a man who had formerly been porter, was in the habit of giving out the stimulants for patients at 7 a.m., and as many of the poor creatures sold their allowance, and others had theirs stolen from them, the nurses had become partially or wholly intoxicated when the doctor reached the house for his morning visit. One of these nurses, by name Charlotte Massingham, had been in supreme authority in the Strand workhouse infirmary for years. She was nearly always muddled, and treated the doctor and his new-fangled notions with supreme contempt. Hearing from Dr. Rogers that his application to the board of guardians for some linseed to make linseed tea for the patients had been granted, Charlotte jumped at least a foot from the ground, and came down slapping her sides and ex claiming, "My God! linseed tea in a workhouse!"

CAUSES OF DEATH.—A strange story reaches us from Germany which seems hardly creditable. Dr. Cornet has published an account on the causes of death amongst hospital nurses, and draws the conclusion that out of every 100, at least 63 die of phthisis. Dr. Cornet maintains that this is because we will not acknowledge the contagious nature of tuberculosis; he warns every nurse to be sure and render innocuous all phthisical sputum by keeping it moist. This is a hint worth remembering by all nurses, and we commend it to their attention.

ANOTHER ABSENT NURSE.—A case of suicide in the nurse's absence took place at Deal last Saturday. Captain Hardy, who, owing to ill-health, had been for some time on half-pay, recommenced official work at Walmer about a month ago. He, however, fell ill again, and in consequence became greatly depressed in spirits. On Saturday he went into hospital, where, during the temporary absence of the nurse under whose care he was, he cut his throat so deeply as to almost sever the head from the body.

DIPHTHERIA.—Nurse Harrison, of the Western Hospital, had an uncomfortable experience last week. A caretaker at Marylebone fell ill, and on Sunday morning sent for the doctor, who pronounced it a case of diphtheria, and sent her to St. Mary's Hospital. Diphtheria no longer being admissible to general hospitals, the woman was sent to the relieving officer, who gave her an admission to the Asylum's Board Hospital. Nurse Harrison arrived in the ambulance for the patient, who was by this time breathing with difficulty. Halfway to the hospital she died, and the case shows how rapid at times is this fatal disease.

DEACONESSES AT UTRECHT.—Further news from the Paris Congress. In the year 1844, four or five ladies in Utrecht, amongst whom we may mention the Baroness de Zuylen de Myevelt, started a plan for opening a small infirmary for women and children, and tried to rouse the interests of their countrywomen in these invalids. And to-day anyone visiting Utrecht might see a complete series of buildings, separated, or rather joined together, by a magnificent garden. Here, under the shade of elms and beeches, on fine summer days, convalescents come and rest, old ladies and children. Sixty or seventy deaconesses, who either have their diplomas or are trying for them, find here a happy home, and a noble use for their talents. After this first work many others followed. At Haarlem, special treatment is given to those suffering from epileptic fits; this is the only establishment of its kind in our country, where, for one hospital for men you may see two for women and children, under the lady deaconesses. Arnhem, Gronnigue, Leuwarde, Wordtect, each have a similar work, proportioned to their population, great or small. A few years ago were introduced among us the services of district deaconesses; they have their office, where they nurse a few sick poor, and where they often give advice for many small miseries, physical and moral. Their consulting hours over, they give a helping hand to families where the illness is not sufficient to warrant the keeping of a sick nurse by the week. The deaconess in Holland, with very few exceptions, gives herself entirely up to the nursing of the sick, refuges, children's schools, the teaching of girls. Many things which in other countries make part and parcel of her work, with us do not enter into it at all. It is not that we do not possess these other works; but the spirit of centralisation which can group several branches under one direction, does not agree with the character of the Dutch. The national spirit tends rather to keeping the integrity of each work to itself, and is jealous of any liberty or individual initiative; the work cannot be said to suffer from it, since it is the human heart and individual love which are the soul of charity.

### Notes from Australia.

*(By Our Own Correspondent.)*

MELBOURNE, June 25.

HOSPITAL accommodation is still the burning question here, and well it may be, for we literally send many applicants for admission to the gaol because we have no vacant beds in the wards. Mr. Derbin Wilder is working hard for increased accommodation, and will, we hope, be soon successful. Some Australians can give splendidly, as Mr. Ormond's bequests show—he has left £113,500 to different charities; still it is not right to ask one or two to support those institutions which benefit all. The suggestion made by Mr. Henry Henty a day or two ago with regard to a means for increasing the fund now being collected in aid of increased hospital accommodation, is worthy of notice. His notion is that the 2d. per lb. which the Government has announced its attention of taking off the tea duty should be retained, and devoted instead to the purposes of the hospital fund. The retention of the tea duty would provide a simple and efficient way of taxing the working classes for their own benefit in a manner which they would scarcely feel. All the working classes drink tea, and there must be few who could not afford for such an object the 2s. 2d. which Mr. Henty estimates would be the amount of the contribution of each individual.

Dr. Rowan has given up his Nurses' Home, and Miss Garlick has taken it. It is situated in St. George-street, East Melbourne. Miss Macartney also has a Nurses' Home, a private venture. The recognised tariff for nurses is £2 a week, or £3 in infectious cases; sometimes more is given. The Church of England Mission here keeps one member—Nurse Ellen—solely to attend on the sick poor. They have lately adopted the rules and services of deaconesses, so that more systematic work may be hoped for. The whole mission only expends £150 a year, so there is room for enlargement.

A home for inebriates has long been talked of here, but while we talked, the Rev. W. L. Morton has been doing. He has started a small "Lodge" at Malvern, where those who desire to try and overcome temptation can be helped in their resolution and kept away from tempters. The lodgers number twelve, and pursue their ordinary work, if they have any, and for those who have lost their situations employment is provided. Many men of education and intelligence fall victims to drink here, and some of the stories told by Mr. Morton are inexpressibly sad. Three doctors give their services gratuitously, and a chemist supplies medicine free of charge.

From the report of the Prince Alfred Hospital, Sydney, of which Miss Catherine C. Downs is matron, I quote the following: "The directors have pleasure in reporting a highly satisfactory condition of the nursing staff. The discipline maintained by the matron, the lectures of the late medical superintendent, and the practical training by the sisters, have enabled the nurses to acquire a high degree of efficiency, and to gain two gold medals, as well as other prizes, at the late Women's Industrial Exhibition. The directors refer with special pleasure to the sympathetic kindness and devotion which the matron and nursing staff have displayed in their trying and arduous work, increased, as it has been, by the great number of serious cases of typhoid. Acting upon the experience of the last five years, it has been decided that the system of selecting probationers should be rendered more definite and stringent; that the course of training for the hospital certificate should be extended to three years; and that arrangements should be made for the delivery of a systematic course of lectures embodying concise teaching upon every subject with which a nurse should be acquainted, the entire course of training being designed to render the nurses practically acquainted with every detail of their work without embarassing them with unnecessary knowledge. The directors are much indebted to those members of the honorary and salaried staff and to others who have kindly undertaken to give these lectures gratuitously. The obligation is enhanced by the necessity for half the nurses having to remain in the wards during lecture time, so that each course has to be delivered in duplicate. To render the course of training complete, Miss Whiteside has been engaged to give a complete series of practical demonstration of cookery for invalids."

At the Homœopathic Hospital the medical staff lately said they were prepared to commence their first term of lectures to pupil nurses. Lectures would be, as usual, on the subjects of anatomy, physiology, and general nursing. The lectures would be delivered by Dr. Teague, Dr. Ray, Dr. Seelenmeyer, and also Dr. Wallace, of Prahran, the newly-appointed member of the medical staff, in the order named.

Some time ago, the Committee of Management of the Hamilton Hospital invited designs for a new building, to cost £10,000, for which a prize of £75 was offered. About thirty architects competed. The judges appointed were Mr. W. H. Marsden, district architect, Public Works Department, and Mr. F. Hammond, C.E. They selected the design of Mr. Beverley Ussher, architect, of Melbourne, and he has been requested to proceed to Hamilton to confer with the committee with regard to the erection of the building.

The Geelong Infirmary deserves some description, and doubtless some of your readers will remember Dr. Arthur W. Marwood, once of Trinity College, Dublin, who took his degree at Edinburgh, and is now resident surgeon at Geelong. Nowhere in all Australia is there a finer site for a hospital and asylum for the aged than at Geelong. The main building of two storeys is composed of three wings, the flanks being added to by spacious verandahs and balconies, where the patients can take air and exercise, having a charming view of the town, and of Corio Bay and the You Yangs. Recently new quarters have been erected at this institution for the nurses' accommodation, and arrangements made for female nurses to take the place of wardsmen where practicable. The committee of the Geelong Infirmary are following the right principle. The Geelong Infirmary and Benevolent Asylum receives a large Government grant added to the local subscriptions and donations. This institution has been in existence since 1854, and has done very good work in administering to the ailments and wants of the sick and suffering poor of the district.

The Immigrants Aid Home does a lot of hospital work. Dr. David Grant is the medical officer, and generally has 214 patients under treatment. One ward at St. Kilda-road was devoted for the whole of the year to the reception of cases suitable for general hospitals who were unable to obtain admission into those institutions owing to the number of typhoid fever cases. This is one of the oldest charities in Melbourne. It was begun in Canna's Town (*i.e.*, before the city was really built), and has gone on increasing, but is yet not large enough for the demands on it.

### Winter Amusements and Remunerative Work.

### AN EXHIBITION OF DOLL NURSES.

THE EDITOR of THE HOSPITAL has much pleasure in offering to nurses prizes to the value of £10 for the three best dressed sets of dolls representing the nursing staff of any hospital. The dolls must not be shorter than four inches nor taller than twelve inches, and must represent the sister, charge-nurse, and probationer of the hospital. Any nurse intending to compete must send her name and address on a post-card, headed "Doll Competition," together with the name of the institution or hospital, the uniform of which is to be faithfully reproduced, to the Editor of THE HOSPITAL, The Lodge, Porchester-square, London, W. *before September 1.* Further particulars will be announced later on, and arrangements will be made for a public exhibition of the dolls where nurses will be able to see the show. The number and amount of the prizes will not be fixed at present in deference to a general wish, and suggestions on this point are invited.

### Physiology for Nurses.

### NO. VI.—ON THE SKELETON. *(Continued.)*

Connected with the head is the spine—a long chain of small bones, each one separate and piled one on the top of the other, and extending in a curve from the head to the bottom of the back. The two uppermost of these bones are different from the others. The topmost of all, on which the skull rests at the back, is larger and broader than the rest, and is called the "Atlas," not because it has any connection with geography, but because it supports the head, and is so called after a supposed man who, the old Greeks and Romans believed, was condemned to bear the weight of the world on his shoulders because he presumed to wage war with certain heathen gods, and, of course, was defeated, and his name was Atlas, and as this bone has to bear the weight of the head, those who gave the bones each their separate name recalled this strange old myth and called that bone "Atlas." The second bone in the spine below "Atlas" is of a very peculiar shape, and is so made to serve a special purpose. It has a projection like a tooth, which acts as a pivot on which the head works, and therefore this bone is called "axis." Sometimes you see thoughtless people try and lift a child up by the head, which is extremely dangerous, and if the head should be lifted a little way even off this peg of the axis it might cause instant death. In the centre of each of the bones of the spine is a round hole, through which you could pass a string and so make a chain of them just as you thread together beads. In the living body the spinal cord, as it is called, runs where your string is—this cord is formed of a great many nerves and is very important to us, and if anything should seriously injure or cut this cord we should die, but we shall hear a great deal more about its wonderfulness when we come to talk of it in its proper place. If the spine was formed only of a number of bones placed one on the top of the other, you would not be able to jump without feeling pain, and perhaps injuring yourself, and if you tripped coming down stairs in a hurry, it might be serious—in fact, if we had to be always thinking about the harm we might do to our spine our lives would be too burdensome to bear. However, to prevent this terrible state of affairs, we have between each bone of the spine a pad of a substance like gristle, and this has exactly the same effect as the buffers of a railway train have, and, just as they prevent the carriages from knocking violently against one another, and making the people inside uncomfortable and shaking them, so the little buffers in the spine prevent us from shaking the delicate spinal cord and doing it harm. Thus the spine is the great support to the body at the back. Here, again, in the curved shape of the spine you get the same principle carried out as you saw in the skull—that is, in both you get greater strength from the curved shape. Attached at the back to the spine, and in front to the breast-bone, are the twenty-four ribs, twelve on each side, but only the seven uppermost are securely fastened, and hence are called "true"; the other five are merely attached to one another, and hence are called "floating" or "false" ribs. These ribs are attached to the breast-bone by pieces of gristle or cartilage, as it is usually called, so as to make them less rigid and stiff, and able to expand when we breathe. You can see what a capital cage the ribs, back-bone and breast-bone make together, and what good protection they give to the lungs and heart, and this cage is further strengthened and completed by another pair of bones of which we have not spoken yet—namely, the two flat shoulder-blades—which are made flat to give plenty of surface for the muscles to be attached to, which help the arms to move, but you will understand how useful and important their shape is when we come to consider how we move our bones. The lower part of the "trunk," or central part of the body, is completed by the pelvis, as the large flat bones are called at the hip. Here again you get bones which are more or less flat because you want some protection for those organs in the abdomen, such as the kidneys, intestines, etc., and you also want room for the very strong muscles which move the legs to be fixed firmly on.

Some of the most wonderful bones you see in the body are those called "long bones" from their shape. These bones are very strong indeed, especially those thigh bones which have to support the weight of the whole body. So wonderfully are they made, and so strong, that when engineers and others who have to put up masonry supports to bear a great weight they find they cannot do better than study very carefully the way in which a thigh bone is shaped and its material is put together. The "long" bones are found entirely in the limbs; each leg and arm has one strong "long" bone in the upper part, and two each in the lower, and each is built up in the same way. If you saw a "long" bone in two down the whole length of it you will see how different it looks at the outside to what it does in the inside. At the outside there is about quarter of an inch of material that looks exactly like ivory—it is so close shaved—this is, in fact, called the "compact" tissue; beyond this the substance of the bone is much more loosely put together: it looks like lace or like the substance of a sponge, and this inside part is called "cancellous" tissue. There is no difference really between the outside or "compact" tissue and the inside or "cancellous" tissue in its actual composition, beyond that in the "compact" tissue the material is squeezed tightly together, and in the "cancellous" it is more loosely put together. In the middle of a "long" bone is a hollow or canal, where the marrow lies. All bones except a few very thin ones that you get in certain parts of the face are formed of these two kinds of tissue, only in the flat bones, like the shoulder-blade and skull, there is a far larger amount of compact tissue, otherwise you would not get strength enough. You can see at a glance what a very powerful bone a thigh bone is. Look at its broad head, set almost at a right angle, and the shaft, or middle part, not made straight and the same thickness all the way down, but with various curves to enable its owner to walk and move with ease and grace, instead of stiffly, as if he had wooden legs. This extra part or head receives the weight, and spreads it throughout the whole length of the bone. If the same weight fell on a bone with a smaller head it would strain it far more, but as it is Nature has made the very best possible arrangement for bearing the weight. At the division of the leg into the upper and lower part a small bone is placed, called the knee-cap, or patella. It is in no way part of the large bones, but is quite apart, and is only kept in place by strong bands. It is almost flat, about 2in. across, and is very strong, made entirely of "compact" tissue. Sometimes when people fall down it snaps across, and then the person who has met with this accident cannot walk, but has to lie up in bed for five or six weeks, and even after that cannot bend the knee for some time afterwards. Women who have to earn their living by scrubbing and have to kneel a great deal get very sore knees, for their little knee-cap bone becomes inflamed and very painful, and all around it becomes red and swollen, and painful, and it is often very serious and keeps them confined to bed for many weeks, and after that very often the knee cannot be used for a long time afterwards, so that they cannot go on scrubbing, and lose their employment. All this trouble, however, could be avoided if they would only always use a cushion with a hole in the middle into which the knee can drop, and not have to bear so much weight. Nature never intended us to kneel for so long, and did not make the knee-cap to bear great weights, therefore if we insist on kneeling for long together we must protect the knee-cap. Here you see the difference between using a bone in the way Nature meant us to do, as when we stand our thigh bone does not become swollen and painful however long we stand. Our feet will give us pain, but that is due to another cause.

*(To be continued.)*

### The Nurse's Bookshelf.

### ANOTHER MANUAL.*

We should like to know on what grounds Miss Dobrée considers herself competent to write a book on nursing, for we can find nothing in the pages before us to justify their publication. The book is supposed to be written for those too poor to employ a trained nurse, or for those who have a prejudice against professional nursing. It consists of six chapters which are neither clear nor concise; much unnecessary information is given, much necessary information is left out. Such sentences as—'This should not be run a chance of,' 'You can be as perfectly cleanly if you choose it,' 'As far as yourself is concerned,' 'When you have reason to believe you or any member of your household has got a fever,' are incorrect; and we should prefer 'read aloud' to 'read out,' 'statistics' to 'statistic,' and 'strips of plaster' instead of 'stripes of plaister.' But these are mere faults of construction and grammar, and might be pardoned were it not that the clumsy sentences teach bad nursing. Here are the directions given for dressing a blister:—"Once the blister has risen, by either means—if a plaister, remove it carefully—give a snip with scissors or prick it and let the fluid run off into a pad of cotton wool." First we would call attention to the punctuation and parenthesis of the above sentence, which are typical of those which prevail throughout the book. Then we would point out that it is well to tell a nurse to puncture the lowest portion of the blister, and to warn her that the doctor will probably expect it to be dressed with some ointment. Perhaps the worst direction given is that on page 124, 'Always see that the thermometer (clinical) is at normal point before you begin to use it.' Apparently Miss Dobrée has never heard that the temperature may sink below the normal point, a serious symptom, which needs attention as much as a high temperature. As the normal height is never mentioned, however, the readers are less likely to endanger their friends' lives by trying to take temperatures. Again, in a case of apoplexy you are told a chief symptom is total unconsciousness, and you are bidden to administer a purgative. The second direction in case of sunstroke is about as incomprehensible as anything we ever read—'Apply cold water; hot water with chill off is better.' Even an Irish Biddy couldn't beat this!

Poisons are divided into Irritants and Neurotics, and in the glossary neurotics are thus explained, 'Medicines which relieve the disorders of the nerves.' It is impossible to point out all the faults connected with poultice-making, fractures, etc., which this book contains; we can only regret that Mrs. Scharlieb should have written an introduction to a work we are perfectly certain she had never read. Such glaring mistakes both in regard to nursing and composition would strike the most casual reader. There are at least half-a-dozen more worthy works on home nursing than this, and though Miss Dobrée has culled liberally from their pages she has only done so to work destruction and confusion. We are quite aware that the perfect work on nursing has yet to be written, but while we can boast such excellent handbooks as Domville's "Manual," Miss Homersham's "Home Nursing," etc., we should prefer that ladies, ignorant apparently of both nursing and composition, should not put their pen to paper to discourse on themes already treated by those whose experience gives them a right to speak.

### Presentation.

Last year Miss C. Lloyd retired from the post of sister-superior of St. John the Divine Sisterhood, which she had held for nearly twenty years. Miss Lloyd, however, remained in the community, and Miss Wilson, the new superior, having to go abroad for her health, Miss Lloyd for some months kindly continued her old duties. Now, however, she has definitely retired, and the sisters and associates of the community have recognised her long and faithful service by the presentation of a handsome address and a purse containing £512. Miss Lloyd proposes to devote the money to the hospital worked by the sisterhood at Poplar.

* "A Manual of Home Nursing." By Louisa Emily Dobree. Swan Sonnenschein. Price 1s. 6d.

### Everybody's Opinion.

[*Correspondence on all subjects is invited, but we cannot in any way be responsible for the opinions expressed by our correspondents. No communications can be entertained if the name and address of the correspondent is not given, or unless one side of the paper only be written on.*]

### CLEAN NAILS.

Doris writes: The article on "Physiology for Nurses" has set me wondering if any of your numerous readers have ever tried to dispense with the nail-brush? It has the same effect (though probably on a smaller scale) as scraping—viz., of enlarging the space for the dirt to collect. I think anyone who can bear to have very dirty nails for a week or two will be repaid by the after comfort, as the dirt will then wash out of the nails as off the hands. I can speak from personal experience of the success of this plan, at least for nurses whose hands are so constantly in water.

### Vacancies.

The following vacancies are announced:

### Matrons.

Central Ophthalmic Hospital, W.C.
Glasgow Victoria Infirmary, £70.
Swansea Hospital, £50.
West Herts Infirmary, £40.

### Nurses.

Birmingham Fever Hospital (several).
Birmingham Orthopœdic Hospital.
Bury St. Edmunds Hospital, £25.
Chelsea Infirmary, £18.
Cheltenham Nursing Institution.
Glasgow Sick Poor Association.
Grantham Hospital.
Hanwell Ophthalmic School.
Kent Nursing Institution.
Kingston Home, Hull.
Liverpool City Hospital, £30.
Maidstone Hospital, £26.
Newcastle-on-Tyne Home.
Nurses' Institute, Jersey, £25.
Protestant Home, Cork.
Rochdale Infirmary, £22.
Seamen's Hospital, £20.
Southport Nurses' Home (several).
Stephen Monckton Home.
Stepney Hospital for Children.
St. Helen's, Lancs., Cottage Hospital, £20.
Stratford-on-Avon Home.
St. Saviour's Infirmary, £20.
Torquay Sanatorium, £30.
Victoria Home, Bournemouth.
West Norwood Institution.
Weston-super-Mare Nurses' Institute, £25.

### District Nurses.

Godalming.

Penzance, £40.

Queen Victoria Institute, Edinburgh.

### Probationers.

Bolton Infirmary.
Brighton Boro' Fever Hospital, £14.
Burnley Victoria Hospital.
Maidstone Hospital.
Royal Free Hospital (paying).
Ventnor Hospital for Consumption, £10.
Workhouse Infirmary Nursing Association.

### Notes and Queries.

To Correspondents.—1. Questions may be written on post-cards 2. Advertisements in disguise are inadmissible. 3. In answering a query please quote the number. 4. If a private answer is desired, a stamped addressed envelope must be forwarded.

Notice.—We must really ask our correspondents to be more particular in enclosing name and address. Constantly we receive queries or letters with no name attached.

### Queries.

(70) *Home Wanted.*—Could you tell me of a home where an aged person could be received for a small sum? She is quite well in health *Mears.* (See answers to query 66).

(71) *Monthly Nurse* asks: What book is the best (for a nurse who wishes to take up monthly nursing) on hints for monthly nursing?

### Answers.

(66) *Home Wanted.*—Four letters have been forwarded to "C. M. H." from the Victoria Home, Leeds, the Woodside Home, Whetstone, and others.

*Somerville Club.*—The editor begs to inform Miss C. that the club is open weekdays and Sundays, lectures being delivered every Friday evening in the season. Address the Secretary, 231, Oxford-street, W.

*Scotch Hospitals.*—"E. C." is advised to procure the *Hospital Annual* from our office, price 2s. 6d. She will there find full particulars of all Scotch hospitals.

*Nurse Bell.*—Sodium ethylate is a liquid; it is rather expensive.

### TRUE HEROISM.

Thou would'st be hero, wait not then supinely
For fields of fine romance which no day brings;
The finest life lies oft in doing finely
A multitude of unromantic things.

